# Long Term Monitoring (2014–2018) of Multi-Mycotoxins in South African Commercial Maize and Wheat with a Locally Developed and Validated LC-MS/MS Method

**DOI:** 10.3390/toxins11050271

**Published:** 2019-05-14

**Authors:** Hannalien Meyer, Zanele Diana Skhosana, Mamsy Motlanthe, Wiana Louw, Egmont Rohwer

**Affiliations:** 1 The Southern African Grain Laboratory (SAGL), Grain Building-Agri Hub Office Park, 477 Witherite Street, The Willows, Pretoria 0040, South Africa; Zanele.Skhosana@sagl.co.za (Z.D.S.); Mamsy.Motlanthe@sagl.co.za (M.M.); Wiana.Louw@sagl.co.za (W.L.); 2 Department of Chemistry, Faculty of Natural and Agricultural Sciences, University of Pretoria, Pretoria 0028, South Africa; Egmont.Rohwer@up.ac.za

**Keywords:** South Africa, multi-mycotoxins, maize, wheat, post-harvest, survey, LC-MS/MS mycotoxin method

## Abstract

Mycotoxins occur worldwide in the major grains, and producers, traders and processors are all challenged to prevent serious health problems for consumers. The challenges originate with pre-harvest fungi infections in the grain fields, increased contamination during improper storage and, finally, the mycotoxin accumulation in commercial food and feed products. Little is known about the multi-mycotoxin occurrence in maize and wheat commercially produced in South Africa. This is the first comprehensive study that reports on the multi-mycotoxin occurrence in South African produced maize and wheat crops after harvest, over four production seasons, in all the production regions of the country. The study was made possible with the development of a fit-for-purpose, cost-effective LC-MS/MS multi-mycotoxin method, validated for 13 “regulated” mycotoxins. A low mycotoxin risk was found in South African produced wheat, with only deoxynivalenol (DON) in 12.5% of the 160 samples at levels well below the 2000 µg/kg South African (SA) regulatory level. It was concluded that aflatoxin B_1_ (AFB_1_) is seldom present in South African produced commercial maize. The concentrations, regional variation and seasonal trends of deoxynivalenol and fumonisins, the two most prevalent mycotoxins, and of zearalenone (ZON), are reported for white and yellow maize in all the production provinces, based on the analytical results of 1400 maize samples. A threefold to eightfold increase in deoxynivalenol mean concentrations in white maize was observed in the main production regions in the fourth season, with 8.9% samples above 2000 µg/kg. A strong correlation was found between higher deoxynivalenol concentrations and the presence of 15-acetyl-deoxynivalenol (15-ADON). The mean fumonisin concentrations were well below the 4000 µg/kg South African regulatory value. A possible shift in the incidence and severity of mycotoxigenic *Fusarium* spp. in the provinces must be investigated. The variations and trends highlight the importance of a continuous monitoring of multi-mycotoxins in South Africa along the grain value chain.

## 1. Introduction

There is a growing awareness of the serious health risks posed by specific mycotoxin contaminations of grain-based food and feed [1,2]. The grain industry needs reliable information on the prevalence of the mycotoxins to develop sustainable mycotoxin management along the food and feed chains. Mycotoxins are toxic secondary fungal metabolites produced by specific fungal species that have a negative impact on global food safety and security, and may be produced when conditions are favourable in the fields during production, during storage and during grain transportation [3,4,5]. The supply chain responsible for food safety is challenged by the fact that mycotoxin contamination is mostly found in localized “hot-spots” in a consignment [4,6]. 

Survey results of mycotoxin contamination in the main staple foods, such as maize, wheat, rice, sorghum, soybeans and cassava, together with the related final food and feed products, are critically important for developing sound practices to reduce mycotoxin contamination along the value chain. Only then can these practices be effectively monitored and improved. The movement of food and feed products across the world, including mycotoxin-contaminated products, highlights the importance of worldwide as well as country- and region-specific surveys on mycotoxin occurrence. The results of such surveys have confirmed that the presence and concentration levels of specific mycotoxins vary among the grain types, production regions and year of production [7,8,9,10]. 

Nowadays, mycotoxins are grouped into “regulated”, “masked” and “emerging” mycotoxins [8], with the list of “regulated” mycotoxins and their maximum allowable levels written in legislations, although not harmonized worldwide. The “regulated” mycotoxins associated with grains generally accepted to have unfavorable health effects in humans and animals include aflatoxins (specifically aflatoxin B_1_ (AFB_1_), a known carcinogen), fumonisins (fumonisin B_1_ and B_2_), ochratoxin A (OTA), trichothecenes type A (T2-toxin, HT-2 toxin) and type B (deoxynivalenol (DON), 15-acetyl-deoxynivalenol (15-ADON), nivalenol) and zearalenone (ZON) [8]. There is not yet enough data on the occurrence and toxicity of the masked and emerging mycotoxins available to date, and therefore no regulations exist for these compounds. 

In South Africa (SA), maize is the main grain produced and consumed as a staple food, followed by wheat [11]. A surplus of maize is produced annually by SA commercial maize producers; the 10-year (2006–2007 to 2016–2017) average maize production in South Africa was 11,096,676 t, with the exception of the 2015–2016 production season, when a severe drought was experienced [12]. In the 2016–2017 production season, an all-time record crop of 9,916,000 t white maize and 6,904,000 t yellow maize were delivered by the commercial maize growers [12]. White maize is a major cereal in South Africa, consumed fresh or processed into milled, cooked or fermented products, and yellow maize is produced mainly for the animal feed industry [12].

**Figure 1:**
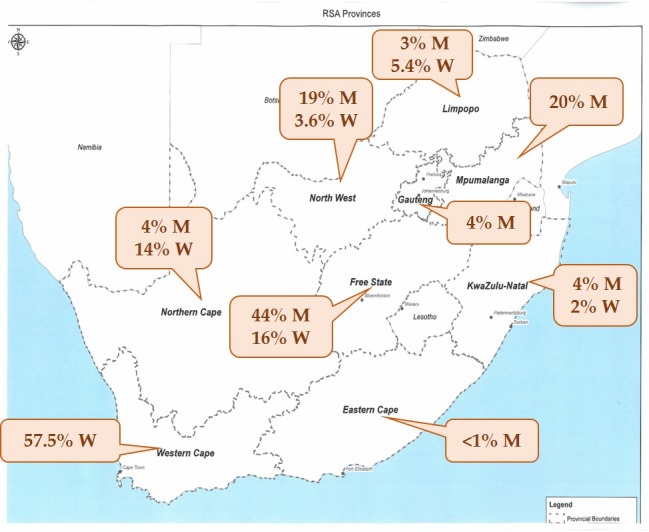
Map of South Africa (SA) showing the production percentages of maize (M) and wheat (W) in nine provinces in 2016–2017. Production for this season was 16.8 million t maize (59% white and 41% yellow maize) and 1.9 million t wheat [12,19].

Although the 10-year average wheat production in SA is 1,826,800 t, SA is a net importer of wheat; during the 2017–2018 season 1,677,162 t of wheat were imported [13]. Wheat contributed 79% to the total winter cereal crop production in the Western Cape winter rainfall region, and was also planted in the summer rainfall regions and irrigation areas of various regions (Figure 1). 

Fumonisin B_1_ (FB_1_) was first reported in South Africa in homegrown maize in January 1990 by Sydenham et al. (1990) [14] in the Transkei, Eastern Cape, where a high incidence of human esophageal cancer was reported [15]. These results led to ongoing research projects in that area of South Africa, with emphasis on the occurrence of fumonisins (FUM) [16], exposure and improvement of storage and cleaning practices of the rural population [17,18]. However, the homegrown maize produced by subsistence farmers contributes to less than 1% of the maize produced annually in SA [12]. Commercial maize is mainly produced in seven of the nine provinces in SA (Figure 1), with three provinces—Free State (44%), Mpumalanga (20%) and North West (19%)—contributing approximately 83% of the annual maize production [12]. 

Aflatoxin B_1_ (AFB_1_), fumonisin B_1_ (FB_1_) and deoxynivalenol (DON) were reported in maize in the SA food mycotoxin studies (from 2007–2016) reviewed by Misihairabgwi et al. (2017) [20]. The reviewed studies focused mainly on small numbers of maize samples collected either in small production areas of subsistence farming [18,20,21] or at research sites in South Africa [22]. In a 2008–2009 maize cultivar study, DON was reported as the most frequently observed mycotoxin, together with 15-acetyl-deoxynivalenol (15-ADON), FB_1_ and zearalenone (ZON) [22]. These multi-mycotoxin results were performed in Germany. Only one study in 2011 focused on commercial maize collected at two feed companies (40 samples) [23]. The only wheat included in the review were DON results of 23 wheat flour samples, analyzed in 2006 [20]. 

Aflatoxins, fumonisins, deoxynivalenol, ochratoxin A and zearalenone were reported in another multi-mycotoxin survey of commercial compound feeds collected at South African feed mills in 2010–2011 (92 samples) [24]. The more-recent annual Biomin surveys, conducted globally, reported mycotoxin results in maize collected in South Africa from 2014 to 2017 [10], and compound feed samples (n = 74) from 2012 to 2015 [8]. A high occurrence of deoxynivalenol, fumonisin and zearalenone, but also aflatoxins and OTA, were reported in the maize samples [10]. With approximately 4.5 million t maize (10-year average) processed in South Africa for the animal industry annually [12], these results clearly may not necessarily represent the status of mycotoxins in animal feed prepared with only SA-produced maize. 

It became important for the wheat and maize supply chain in South Africa to gain insight into the occurrence and prevalence of at least all the “regulated” mycotoxins in SA commercially produced crops. The industries realized that multi-mycotoxin results of a representative selection of the annual crop quality survey samples would give a complete overview of the multi-mycotoxin prevalence in SA maize and wheat, post-harvest before storage. For these surveys, approximately 1000 maize samples and 350 wheat samples were collected annually when the producers delivered their harvest to the grain storage facilities [12,19].

When this study was commenced in 2014, only aflatoxin B_1_ was regulated (since 2004) in SA, in various commodities for human consumption [25]. For farm feeds, maximum levels are written in SA legislation for aflatoxin B_1_, deoxynivalenol, fumonisin B_1_, ochratoxin A and zearalenone [26]. Because only AFB_1_ was regulated for human consumption of grains, aflatoxin testing was often presented as the only necessary mycotoxin test to approve grains fit for human consumption. For this monitoring study, the decision was made to include at least the mycotoxins with maximum levels listed for grains in the Codex general standard for contaminants and toxins in food and feed [27], and those with regulated or guidance values in the European Union [28,29]. As pointed out by Stroka et al. (2016) [30], social considerations may both help and hinder the compound selection process. The compound 15-acetyl deoxynivalenol (15-ADON), considered as a co-contaminant with DON [31], was of interest to local research groups [22]. 

A total of 13 mycotoxins were identified to be monitored in the SA maize and wheat: aflatoxins (B_1_, B_2_, G_1_, G_2_) DON and 15-ADON, fumonisins (B_1_, B_2_, B_3_), OTA, T2-toxin, H-2-toxin and ZON. Fortunately, by that time, the development of LC-MS/MS instrumentation provided a robust routine means for the simultaneous analysis of organic compounds at ng levels in complex food commodities. This allowed commercial analytical laboratories to develop LC-MS/MS methods for the analysis of multi-mycotoxins in different food commodities. The establishment of a test facility in SA conducting multi-mycotoxin analyses was necessary to obtain a long-term, representative picture of the multi-mycotoxin status of maize and wheat produced in SA. 

The extraction methods used for the determination of single compounds or groups of toxins with HPLC techniques were initially considered for the development of multi-mycotoxin extraction methods in grains, cereals and feed products [32,33]. It was soon realized that the choice of solvents to be used for extraction was complicated by the wide range of polarities, the concentration ranges of the mycotoxins and the complex product matrices. Marschik et al. (2013) [34] compared the yield of fumonisins in various naturally contaminated, unprocessed and processed maize matrices with five different multi-mycotoxin-based extractants, and concluded that the extraction solvent mixture—methanol/acetonitrile/water (1:1:2) with a 15-min extended extraction time—was the most appropriate for fumonisins in maize. Our laboratory compared three extraction methods: (i) methanol/water (4:1, v/v) extraction (1 min blend); (ii) double-extraction first with acetonitrile/water/formic acid (80:19.9:0.1, v/v/v) (60 min shake) followed by acetonitrile/water/formic acid (20:79.9:0.1, v/v/v) (30 min shake) [35]; and (iii) methanol/acetonitrile/water (1:1:2) (1 min blend, 15 min shake) [34]. It was concluded that complete extraction of FB_1_, FB_2_ and FB_3_ and DON in highly contaminated maize was achieved with longer extraction times and an increased amount of water added to the extraction solvent mixture. The mycotoxin results of the methanol/acetonitrile/water (1:1:2) and the double extraction method compared well, but a 90-min total extraction time in a routine laboratory conducting large numbers of samples in the main grain season is not justifiable if it is not of the utmost necessity to report correct results. 

Purification of the extracts with clean-up columns is essential for HPLC analysis, but these clean-up procedures were not applicable for the simultaneous analyses of different groups of mycotoxins. Most of the LC-MS/MS multi-mycotoxin methods followed the so-called “dilute-and-shoot” approach, because with the high sensitivity of the LC-MS/MS it is possible to inject diluted sample extracts with a 20-fold smaller sample load (compared to HPLC analyses) and still reach the required limit of quantitation defined in Section 5. 

For the calibration of the LC-MS/MS, matrix-matched standards and stable isotope-labelled standards were considered for the accurate quantification of mycotoxins in various commodities [35]. The unavailability and costs of all the isotope-labelled compounds and the challenges to import them into SA were carefully considered. To keep the monitoring costs as low as possible without compromising the quality of the test results, a decision was made to use matrix-matched standards for the method validation, as described in Section 5. 

The comprehensive data sets of 13 mycotoxins reported in this four-year monitoring project of SA-produced maize and wheat collected after harvest from the 2013–2104 to 2016–2017 maize production seasons and the 2014–2015 to 2017–2018 wheat production seasons are presented in this study. In total over four years, 1400 maize and 160 wheat samples were analyzed in the test facility that was successfully established in SA to routinely conduct multi-mycotoxin analyses on grains and related grain-based products and feeds. The results cover all the commercial maize and wheat production areas in SA. 

## 2. Results

### 2.1. Mycotoxins in SA Commercially Produced Wheat

Of the thirteen mycotoxins monitored, only deoxynivalenol was found in South African wheat. The DON contaminations were low, as shown in Table 1, with the highest incidence rate of 17.5% (7/40) observed in the 2017–2018 season. The mean concentrations varied between 202 and 397 µg/kg, and the highest concentration (593 µg/kg) was found in a wheat sample taken from 2015–2016 production. The samples contaminated with DON came from different production regions. 

### 2.2. Mycotoxins in SA Commercially Produced Maize 

#### 2.2.1. Mycotoxins Found and Not Found

No T2-toxin, HT-toxin, OTA and almost no aflatoxins were found in the post-harvest maize samples (350 × 4) collected over four production seasons, from 2013–2014 to 2016–2017. Only in the 2014–2015 production season, total aflatoxin contamination was reported in three white maize samples from three different regions in the North West province. One sample was contaminated with 6 µg/kg AFB_1_, one sample with 26 µg/kg AFB_1_ and 13 µg/kg AFB_2_ and the third sample had 48 µg/kg total aflatoxins, containing 33 µg/kg AFG_1_, 9 µg/kg AFB_1_ and 6 µg/kg AFG_2_. 

**Figure 2:**
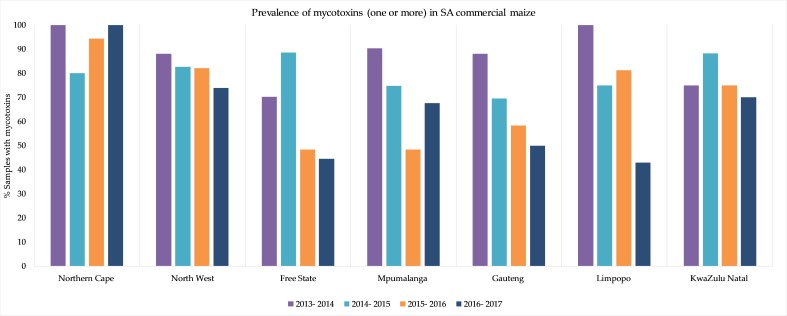
Prevalence of mycotoxins (one or more) in commercial maize (white and yellow combined) found in seven production provinces (as shown in Figure 1) over four seasons from 2013–2014 to 2016–2017.

The mycotoxins found in the SA commercial maize were DON, 15-ADON, fumonisins (FB_1_, FB_2_, FB_3_), and zearalenone (ZON), with at least one of these mycotoxins found in 83% of the samples (white maize and yellow maize) in the first season, and 80%, 63% and 62% in the successive seasons. Deoxynivalenol and fumonisins co-occurred in approximately 26% of the samples. The occurrence in the seven different provinces showed a notable variation between the provinces and seasons (Figure 2). The Free State province that produces approximately 44% of commercial maize annually had a smaller incidence rate compared to Mpumalanga (20%) and North West (19%) [12]. These variations are further elaborated on in the reporting of the individual mycotoxins’ occurrence patterns, mean concentrations (mean of the positive samples) in the different provinces and concentration distributions per season.

#### 2.2.2. Deoxynivalenol and 15-acetyl-deoxynivalenol Occurrence

Deoxynivalenol was present in commercially produced maize in all four seasons in six of the seven provinces in South Africa. In the seventh province, Limpopo, DON was found only in both the white and yellow maize produced in the first season (2013–2014). Sixty-nine percent of the samples was contaminated with DON in the first year, and 41%, 23% and 37% of the samples in the following three seasons. The percentage samples with DON present in white and yellow maize showed the same trends in the first three seasons, but, in contrast with the 54% of white maize samples that contained DON in the fourth year, only 19% of the yellow maize sampled was contaminated with DON. 

**Figure 3:**
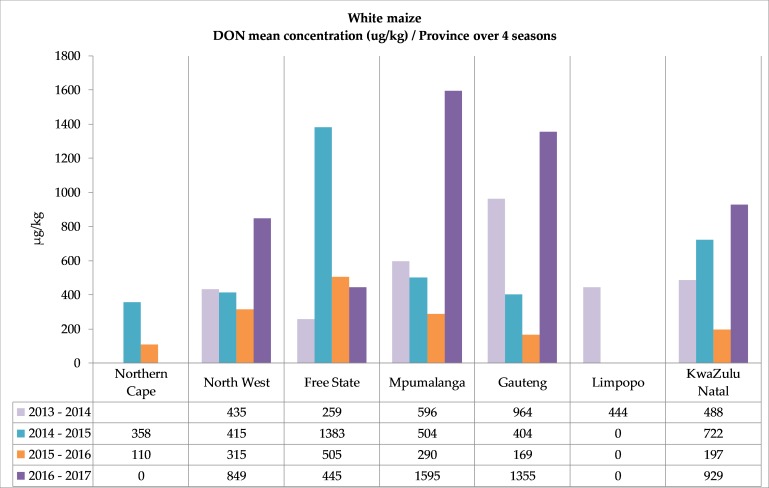

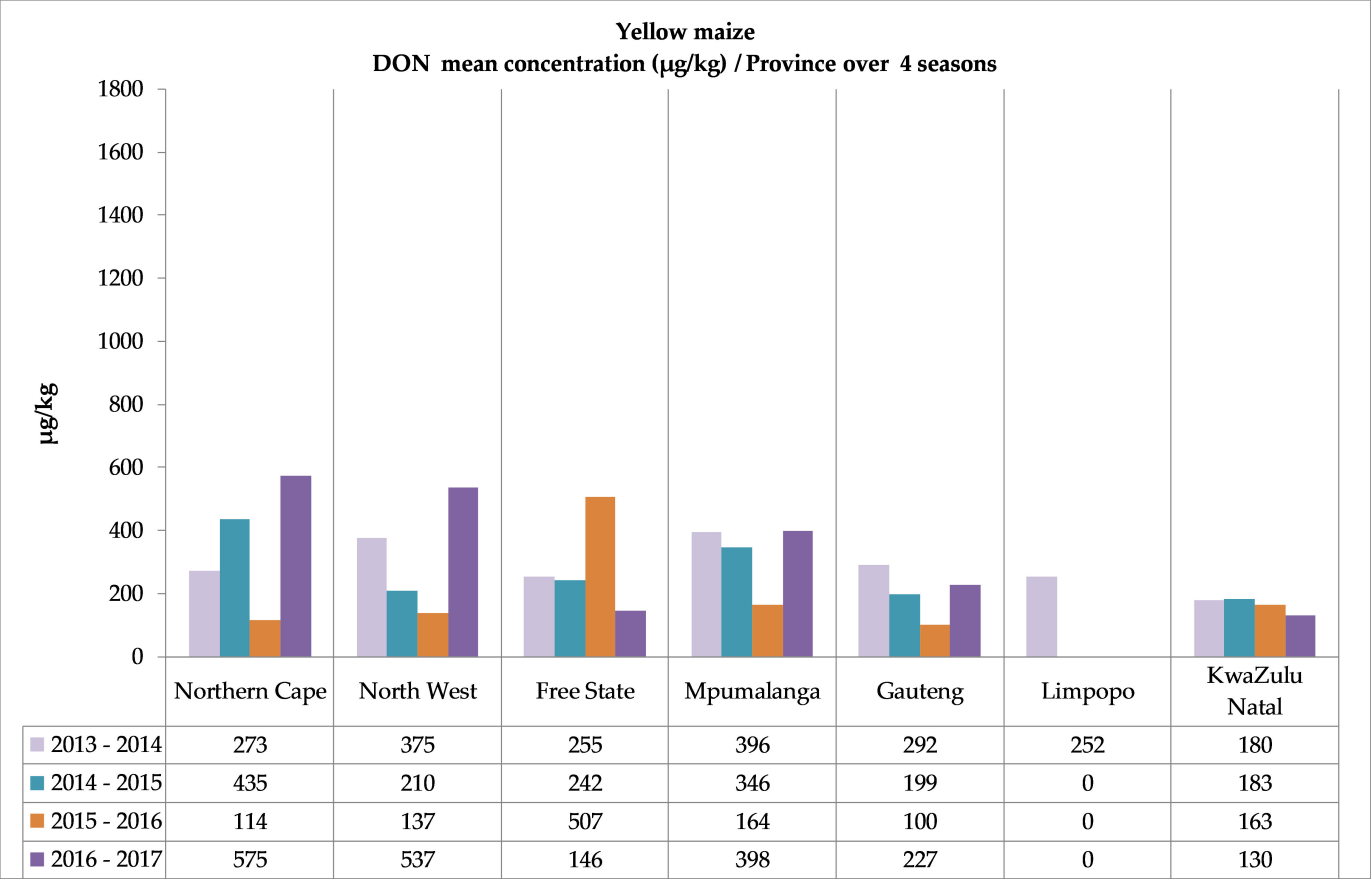
Summary of DON mean concentrations (average of the positive samples) in white and yellow maize. (**a**) White maize mean DON concentrations over four seasons in seven provinces. (**b**) Yellow maize mean DON concentrations over four seasons in seven provinces. Mean values based on positive samples. See Supplementary data for corresponding numbers of samples and mean and maximum values.

The annual mean DON concentrations in the white and yellow maize in the seven different provinces (Figure 3) showed higher DON concentrations in white maize in most provinces in almost all the seasons. The highest mean levels of DON in white maize were reported in the 2016–2017 season in four provinces (849 µg/kg in North West, 1595 µg/kg in Mpumalanga, 1355 µg/kg in Gauteng, 929 µg/kg in KwaZulu-Natal), although only 54% of the white maize samples contained DON. In agreement with this increase in mean concentration levels, the DON concentration range distribution in white maize, as summarized in Table 2, showed that the highest incidence of DON in white maize above the 2000 µg/kg regulated level for human consumption was also recorded in 2016–2017 (8.9% samples; max. 7698 µg/kg). These samples were collected in four different provinces (North West, Mpumalanga, Gauteng and KwaZulu-Natal). A different DON concentration pattern was observed in white maize produced in the Free State, where the highest mean concentration (1383 µg/kg) was reported in the second year of the survey and the individual sample with the highest DON concentration (9736 µg/kg, 2014–2015) was found. 

In yellow maize, the mean DON concentrations were lower and showed a smaller variation between seasons compared to white maize. The highest mean levels were reported in the Northern Cape (575 µg/kg 2016–2017) and North West (537 µg/kg, 2016–2017) (Figure 3). Only in 2013–2014 were 0.5% of the 185 yellow maize samples reported with DON > 2000 µg/kg (Table 2). 

**Figure 4:**
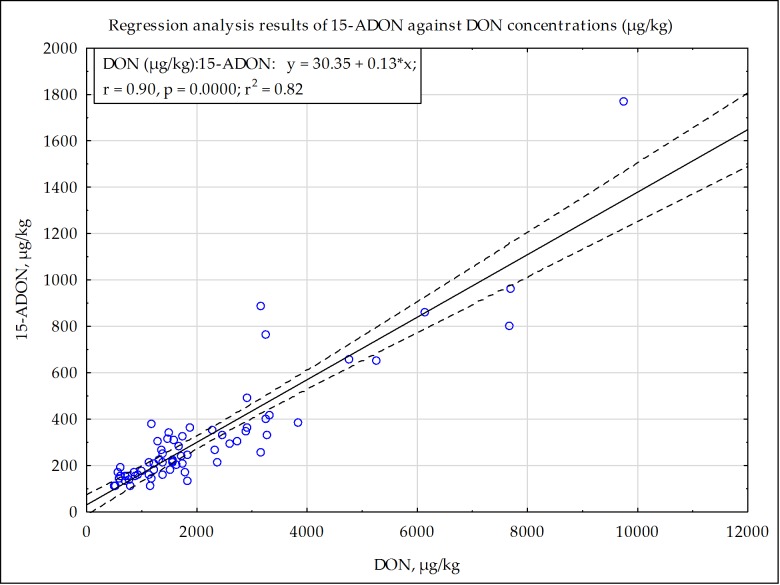
Correlation between DON and 15-ADON concentrations in 121 maize samples with DON > 500 µg/kg and 15-ADON > 100 µg/kg (LOQ).

As expected, 15-ADON was only found in maize that contained DON. Over all four seasons, 15-ADON co-occurred in 85.8% of 141 samples that were contaminated with more than 500 µg/kg DON. The 15-ADON concentration ranged from 100 µg/kg (LOQ) to 964 µg/kg, with the only exception being 1768 µg/kg 15-ADON found in the white maize sample with the highest DON concentration (9736 µg/kg), reported in 2014–2015 in the Free State. A linear relationship was shown (Figure 4) with a regression analysis of a stratified randomized subsample (using Statistica 13.5.0, 95% confidence interval) of all the samples (121) with DON > 500 µg/kg and 15-ADON > 100 µg/kg. The correlation coefficient r = 0.90 with coefficient of determination r^2^ = 0.82 shows a usable correlation between 15-ADON and DON concentrations.

#### 2.2.3. Fumonisin Occurrence

Fumonisins were found in maize produced in all the provinces; 41% of the samples were contaminated in the 2013–2014 season, with 56%, 57% and 44% of the samples contaminated in the consecutive years. The white maize and yellow maize had almost the same incidence rates over the four seasons. 

The highest levels of FUM (total = FB_1_ + FB_2_ + FB_3_) in maize were found in the third season (2015–2016), namely, 6865 µg/kg in white maize and 11,347 µg/kg in yellow maize in samples from the North West province (Supplementary Tables S1 and S2). However, in the three other seasons, the highest FUM levels were found in other provinces. In the first season, the maximum concentration (2927 µg/kg) in a white maize sample was from the Free State. In the second season, 1809 µg/kg was found in the Gauteng province, and 3913 µg/kg was found in the fourth season in the Northern Cape province. In yellow maize, the samples with the highest FUM levels were found in Mpumalanga (5357 µg/kg in the first season, and 6059 µg/kg in the fourth season) and in the Free State (3382 µg/kg in second season) (see Supplementary Tables S1 and S2).

**Figure 5:**
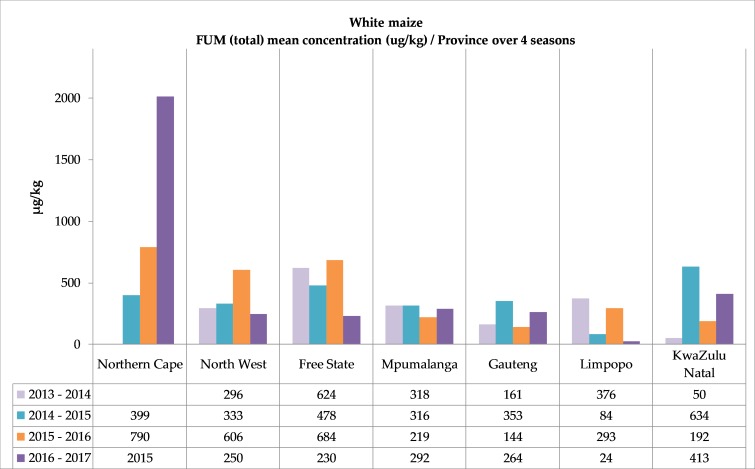

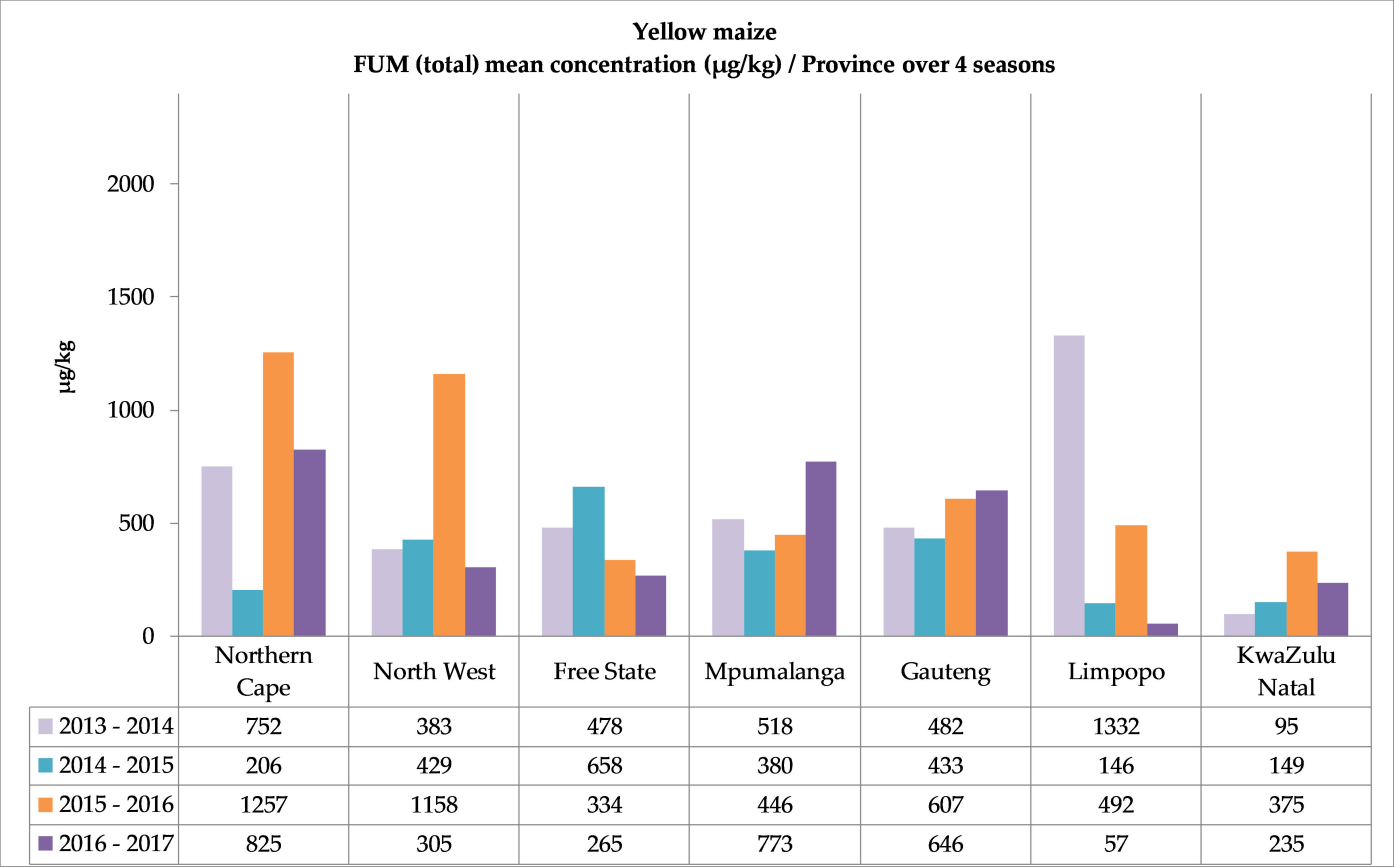
Fumonisin (FUM) occurrence in white and yellow maize. (**a**) White maize mean FUM concentrations over four seasons in seven provinces. (**b**) Yellow maize mean FUM concentrations over four seasons in seven provinces. Mean values based on positive samples. FUM (total) = FB_1_ + FB_2_ +FB_3_. See Supplementary data for corresponding numbers of samples and mean and maximum values.

The overall mean concentration levels of the different seasons calculated separately on all the white and yellow maize samples showed an increase in white maize from 304 µg/kg in the first season to 498 µg/kg in the fourth season. The mean concentrations of the yellow maize varied slightly—577 µg/kg in 2013–2014, and 343, 563 and 444 µg/kg in the following three seasons. The mean FUM concentrations in the different provinces also showed a variation (Figure 5). In 2015–2016, in white maize, mean concentrations > 600 µg/kg were measured in three provinces, including two of the highest production regions, North West and the Free State. In Northern Cape, although a small contributor to the annual white maize supply in the last three seasons, the mean concentration in 2016–2017 was 2015 µg/kg (n = 2/3) and an individual white maize sample had 3913 µg/kg FUM. 

The FUM concentration range distribution, summarized with an emphasis on the regulated values for human consumption (Table 3), showed its highest incidence in 2015–2016, where 0.6% white maize was contaminated with FUM (total) above the maximum allowable level of 4000 µg/kg (max. 6865 µg/kg), and 2.6% white maize samples had concentrations between 2000–4000 µg/kg. In the last two seasons, 1.0% and 1.2% of the yellow maize samples had FUM > 4000 µg/kg (max. 11,347 µg/kg and max. 6059 µg/kg), respectively. 

#### 2.2.4. Zearalenone Occurrence

**Figure 6:**
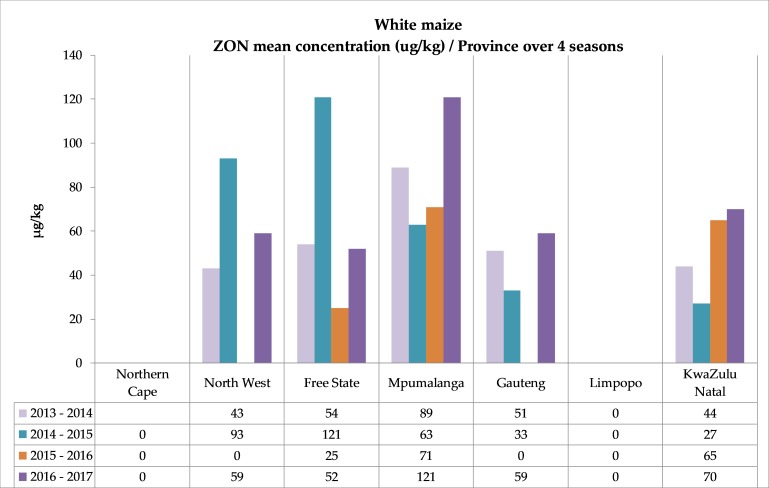

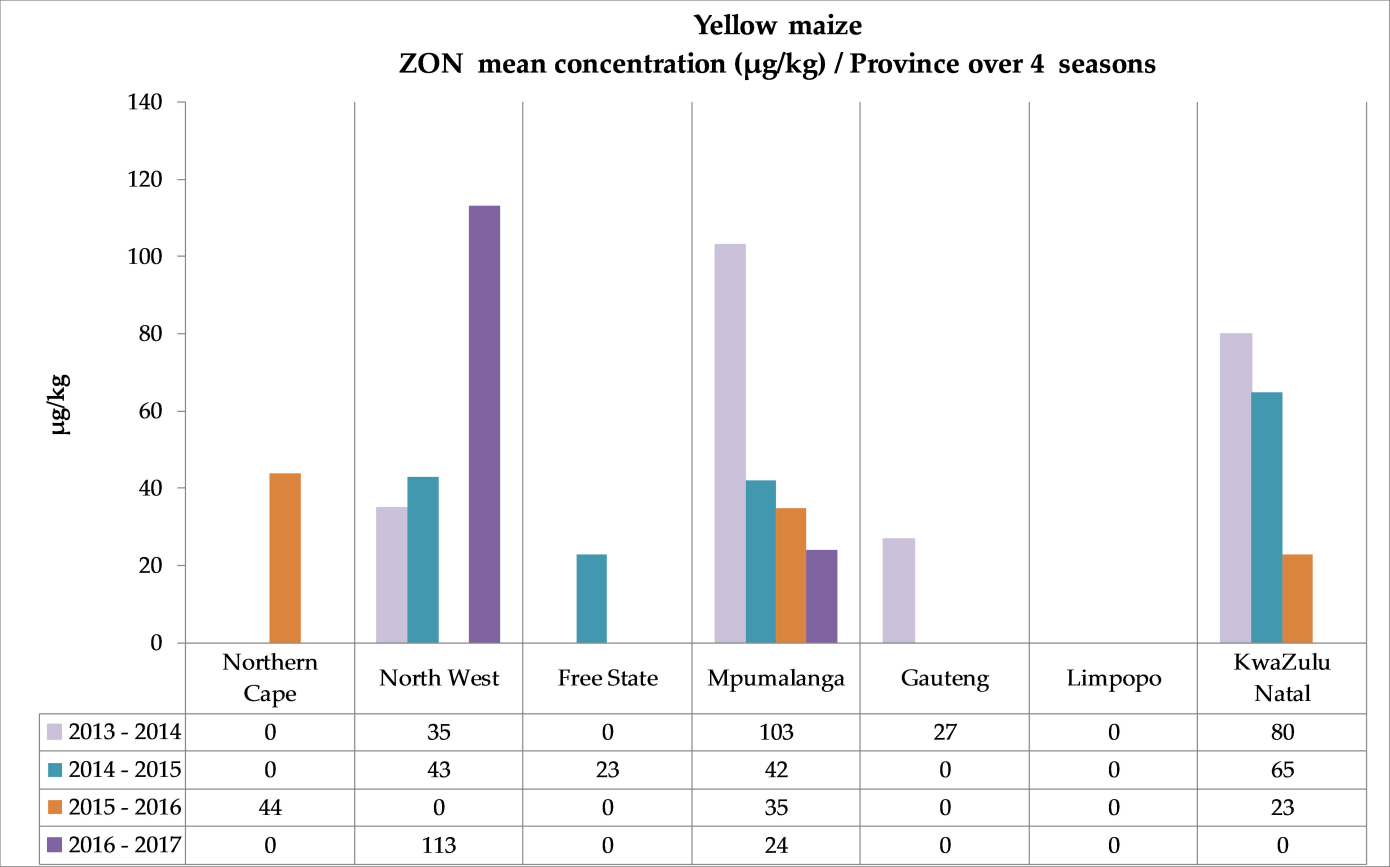
Zearalenone occurrence in white and yellow maize. (**a**) White maize mean ZON concentrations over four seasons in seven provinces. (**b**) Yellow maize mean ZON concentrations over four seasons in seven provinces. Mean values based on positive samples. See Supplementary data for corresponding numbers of samples and mean and maximum values.

Overall, less than 10% of all samples were contaminated with zearalenone. In the second season, the highest prevalence (14%) was observed in white maize. None of the white maize in the Northern Cape and Limpopo provinces contained any ZON, but in three provinces—Free State, Mpumalanga and KwaZulu-Natal—zearalenone was found in all four seasons (Figure 6). The percentage samples with concentrations in the range 100–350 µg/kg increased from 1.2% in 2013–2014 to 4.5% in 2016–2017, and in the first and fourth seasons only one individual sample contained more than 350 µg/kg ZON (Supplementary Table S1).

The yellow maize ZON contamination reduced from 10% of the samples in 2013–2014 to only 1% in the fourth season. The occurrence pattern (Figure 6) showed no zearalenone in Limpopo, only in one season in Northern Cape (2015–2016), Free State (2014–2015) and Gauteng (2013–2014), in three seasons in North West and KwaZulu-Natal and in all four seasons in Mpumalanga. Only 0.6% of yellow maize had ZON concentrations in the range 100–350 µg/kg in 2016–2017, and only one individual sample in this four-year study contained more than 350 µg/kg (354 µg/kg, Mpumalanga, 2013–2014) (Supplementary Table S2).

## 3. Discussion

The results of the 13 mycotoxins monitored over four seasons gives a South African perspective on the mycotoxin status of commercial maize and wheat produced in the country for the first time. In total, 1400 maize (350/season) and 160 wheat (40/season) samples, collected after harvest from commercial producers in South Africa from four production seasons were analyzed. The mycotoxins reported confirmed some but also contradicted results reported in other studies on maize and wheat samples collected in South Africa. 

### 3.1. Wheat 

Overall, South African-produced wheat has a low incidence of only DON, which was observed in only 12.5% of the wheat crop. It must be noted that SA imports wheat to supplement the local demand (934,765 t in 2016–2017) from mainly the Czech Republic, Germany and Russian Federation [19]. When this study’s results are compared with the results of the European wheat summary reported by Stanciu et al. (2015) [9], DON contamination as high as 100% was reported in wheat, including from Czech Republic, Lithuania, Romania and Slovakia. The mean and maximum DON concentrations of SA wheat summarized in Table 1 are notably lower than the maximum DON concentrations reported in the European wheat summary—a concentration as high as 10,000 µg/kg DON was reported in the European results [9]. The SA DON annual mean values are also lower than the annual mean DON concentrations reported in durum wheat at farm level across USA and Canada [7]. No T2-toxin, HT-2 toxin or zearalenone were reported in the SA-produced wheat samples compared to the notable percentages of European contaminated wheat samples [9]. The mycotoxin-free wheat offered by the South African producers should be acknowledged as a valuable commodity.

### 3.2. Maize

#### 3.2.1. Mycotoxins Not Found

The absence of AFB_1_, being a potent carcinogen [37], and the three related aflatoxins in SA commercially produced maize in all seven maize production provinces, is an enormous food and feed safety advantage for the commercial maize producers in South Africa. This non-occurrence was also reported in the Biomin study (2014–2017), where only 0.7% of the 282 maize samples collected in SA were above 5 µg/kg aflatoxins [10]. Contrary to our results, in one assessment conducted in 2010 at two feed mills in KwaZulu-Natal [23], AFB_1_ was reported in 70% of the 40 maize samples collected. It must be noted that different results, obtained with thin-layer chromatography and high-performance liquid chromatography, were reported [23]. In all the studies conducted on maize produced in rural areas by subsistence farmers, high concentrations of AFB_1_ were reported (max 133 µg/kg) only in Limpopo and Mpumalanga [21]. 

#### 3.2.2. Effect of Maize Class, Production Year and Production Province

At first glance, the notable decreases observed in the percentages of maize samples (both white and yellow) contaminated with at least one mycotoxin in the four-year period in most of the provinces (Figure 2) look promising. The decreases mostly occurred because the percentage samples contaminated with DON decreased in yellow maize from 65% in the first year, to 19% in the fourth year, and in white maize from 74% to 54%, respectively. However, the mean DON concentrations in white maize increased sharply (three- to eight-fold) in the fourth season in four provinces that contributed to approximately 42% of the white maize production, including North West and Mpumalanga, two main white maize production provinces [12]. This increase in the mean concentrations were caused by a sharp increase in the number of individual white maize samples with high DON values. In the fourth season, 17.8% of the white maize samples (32 samples) contained DON > 1000 µg/kg (Table 2). Although this increase was reported in four provinces, 20 of these samples were from all the regions in the Mpumalanga province where approximately 11% of white maize was produced in 2016–2017 [12]. 

The yellow maize DON and ZON levels in Mpumalanga yielded a different result in the 2016–2017 season. The sharp increases observed in both DON and ZON in white maize were not present in the yellow maize, although yellow maize production in Mpumalanga was more than that of white maize production in the province [12]. White maize and yellow maize are produced under the same environmental conditions (rain fall and temperatures), and most probably also with the same agricultural practices (tillage, crop rotation, pesticides), yet only two yellow maize samples had DON > 1000 µg/kg. The mean yellow maize DON concentrations (398 µg/kg) stayed almost the same over four years of production (Figure 3), and the ZON mean concentrations decreased over the four seasons. These differences were observed in the same production areas for white and yellow maize, and therefore cultivars planted may play an important role in DON production in the fields. In a study conducted in New Zealand, hybrid choice was identified as major factor, apart from seasonal effects, affecting the incidence and concentration of DON in harvested maize [38]. ZON was not reported in the Limpopo province, and DON was reported only in the first season of this study. Differences in the responses of maize cultivars to infection with *Fusarium graminearum* were previously observed in the cultivars investigated in 2007–2009 [39]. An investigation of *F. graminearum* distribution and infection in maize samples in our study will give more insight into the fungal resistance of white and yellow maize cultivars, as well as into in the observable shift of higher DON and ZON levels found in white maize in the three other provinces (North West, Gauteng and KwaZulu-Natal). 

For the first time, the co-occurrence of DON and 15-ADON in all commercial production areas of SA were monitored, and a correlation is reported. Compared to the DON and 15-ADON results reported in the 14 cultivar trials [22], higher maximum DON levels were reported in our study with a strong correlation between 15-ADON and DON concentrations. 

Regarding fumonisins (FB_1_, FB_2_ and FB_3_), this comprehensive survey confirms the presence of fumonisins in SA maize—in terms of prevalence, approximately 50% of the samples overall were contaminated with fumonisins. The yellow maize mean concentrations reported in Mpumalanga, Gauteng and KwaZulu Natal showed an increasing trend over the three seasons. In the Biomin study (2014–2017), 80% of the maize samples contained fumonisins, and the maximum concentration reported was higher (16,932 µg/kg) [10]. The Biomin study did not mention the maize class (white or yellow) of the samples included in their study.

In contrast to the mentioned DON contamination differences between white and yellow maize, only slight variances were observed in the fumonisin results. Most of the mean FUM concentrations in all seven provinces in each season were less than 500 µg/kg (Figure 5). In all four seasons in Northern Cape and in 2015–2016 in North West, larger FUM concentration differences between white and yellow maize were observed, and the mean white maize FUM concentration increased three-fold (Figure 5). These trends need to be investigated. Jansen van Rensburg et al. (2015) [39] observed a tendency between fumonisin contamination and mean and maximum temperatures in a study conducted in 2007–2009. 

In comparison with fumonisin concentrations reported in previous smaller, localized studies, the Eastern Cape good homegrown maize produced by subsistence farmers in the multi-year study (1997–2003) reported higher incidence rates and mean and maximum concentrations [16]. In the 2007–2009 study, where maize samples were collected from 14 cultivar trials, 62% of the samples were contaminated with FB_1_ and FB_2_ [22], with maximum values in accordance with the highest FUM concentrations reported in individual white and yellow maize samples in our study (Supplementary Tables S1 and S2). In the comparison of fumonisin-producing *Fusarium* spp. and the fumonisin contamination of six cultivars in different provinces from 2007–2009, correlations suggested that cultivars reacted differently to different environmental/inoculum disease potentials [39]. 

In three seasons, ZON was found in 13%–14% of white maize, while in 2015–2016 it was only found in 6%. The yellow maize contamination incidence decreased over the four consecutive seasons from 10% to 1%. None of the mean ZON concentrations (both white and yellow maize) reported in the different provinces over four consecutive years were higher than 121 µg/kg. These results contradict the 47.1% zearalenone incidence rate (145 of 308 samples) and maximum concentration of 6276 µg/kg reported in the Biomin study (2014–2017) [10]. Unfortunately, the Biomin sampling plan was not described in detail to investigate if this comparison is valid with regards to the sampling point in the grain handling chain [8,10]. It must also be noted that the Biomin samples were analyzed with different analytical techniques, including HPLC and LC-MS/MS and possibly different LOQs. The ZON results reported in the 14-cultivar trial [22], and the positive mean ZON contamination in moldy maize (111 and 135 µg/kg) from the rural subsistence farmers in Eastern Cape reported by Shephard et al. (2013) [18] compared with the ZON results of this study. 

### 3.3. Regulated Mycotoxins and Maximum Allowable Levels

Based on the mycotoxin results of the first two years of this monitoring project, South Africa published an amendment in 2016 to regulate DON in cereal grains and FB_1_ + FB_2_ in maize intended for further processing (corresponding with the Codex standard [27]) [36]. DON is now regulated in SA in cereal grains (wheat, maize and barley) for human consumption, with 2000 µg/kg the maximum allowable concentration in grain intended for further processing [36]. Further, monitoring of DON prevalence in wheat over four consecutive production seasons (1,910,000 t in 2016–2017 [19]), as shown in Table 1, confirms that using South African wheat for wheat flour and related processed food does not pose a high risk for consumers. 

Individual maize samples, both white and yellow, with FUM concentration levels above the regulated maximum allowable levels of 4000 µg/kg (FB_1_ + FB_2_) and DON >2000 µg/kg in unprocessed maize, were reported every season in different regions (Supplementary Tables S1 and S2). The sharp increase in the percentage of white maize with DON levels notably higher than 2000 µg/kg reported in this study (Table 2) confirmed the importance of mycotoxin analysis in the grain value chain. To achieve acceptable low levels in all unprocessed maize for further processing, grain storage facilities should take mycotoxin levels into consideration when blending the grain. The results reported confirm that grain handlers should know the mycotoxin status of their stocks.

To assist maize growers to reduce yield loss and mycotoxin contamination due to fungal infections, based on the results of this study, the fungal incidence and severity of commercial maize produced in all provinces must be determined. Shifts identified over production seasons will have an impact on the selection of cultivars, crop rotation programs and agricultural practices. With the fungal occurrence known, region-specific maize producers may be advised on the selection of resistant cultivars and best agricultural practices.

## 4. Conclusions

This is the first comprehensive study that reports on the multi-mycotoxin occurrence in commercially produced wheat, white maize and yellow maize in South Africa. The study was conducted over four consecutive seasons in all the production provinces in South Africa by collecting samples at grain storage facilities when delivered by producers. The establishment of an accredited test facility for the multi-mycotoxin analyses, with a capacity to analyze 350 maize and 40 wheat samples every year at the end of the harvest seasons, enabled the present study, and will be of utmost value for future related projects. A validated LC-MS/MS method (ISO 17025 accredited) with the aim of providing an affordable, fast and sustainable commercial service in South Africa, was successfully developed for the analysis of the 13 main mycotoxins in grain. The accuracy of the method was confirmed with international proficiency tests and interlaboratory comparisons requested by European companies involved in the grain industry in SA. 

The results showed for the first time:

- the presence of only deoxynivalenol (at low concentrations) in SA wheat;
- the absence of aflatoxin B_1_ in maize produced commercially in SA in all the regions;
- the concentrations, regional variation and seasonal trends of deoxynivalenol, fumonisins and zearalenone in white and yellow maize;
- the low prevalence of fumonisin-contaminated maize above the 4000 µg/kg regulated value for unprocessed maize for human consumption;
- an increase in the percentage of white maize produced with DON above the 2000 µg/kg regulated maximum level.

The outcome of the study underwrites various aspects of the Mycotoxin Charter launched in 2018 as part of the European Union funded project MycoKey [40]. The safety of the domestic consumers of maize and wheat products was improved. Based on the results of the first two seasons, SA expanded the regulated mycotoxins in grain to include maximum allowable levels for fumonisins in maize and DON in cereal grains intended for further processing, as well as maximum levels in related processed food products ready for human consumption [36]. The enforcement of this amended regulation was possible only after the successful establishment of the analytical test facility in SA. 

The maize and wheat producers in South Africa are now well positioned (with ongoing monitoring) to arrive at well-informed decisions, and to suggest solutions for mycotoxin problems as they arise in fields. The trends reported will guide specific research to establish rational decisions/solutions for addressing potential mycotoxin problems in fields. Grain storage facilities may develop new strategies for the correct handling and mixing-in of contaminated maize based on the occurrence and trend results.

Mycotoxins will always be the unwanted needle in the haystack, and in spite of all the information available about the negative health effects caused by mycotoxin-contaminated staple foods, reoccurring outbreaks of food and feed poisoning in Africa do occur. This study confirmed that it is possible for countries in Africa to establish an affordable test facility to monitor the staple grains produced in order to protect their consumers.

## 5. Materials and Methods 

### 5.1. South African Commercial Maize and Wheat Samples Collection

Maize is planted in SA in summer, ideally in November–December, and harvested in autumn the next year. Wheat is planted in winter, from April to July depending on the region, and harvested from October to December. The maize and wheat samples for the crop quality surveys, post-harvest to pre-storage, are collected when the crop is delivered by the commercial producers at the commercial grain storage facilities [12,19]. The grain handlers take a representative sample from each consignment for grading purposes and, after grading, approximately 100 g of each sample is placed in corresponding bins (50–100 kg) as a subsample, according to the class and grade awarded per silo bin at each silo. Each full composite bin is mixed well before a 3-kg subsample is sent to the laboratory for crop quality analyses [12,19]. The samples received are composite samples representing maize and wheat per class and grade, produced in all the commercial production regions in South Africa [12,19]. 

Within each production season, approximately 1000 maize samples are selected, proportionally representative of all the production regions for both white and yellow maize, for crop quality analyses. Of these, 350 maize samples (500 g each) are selected each season for mycotoxin analyses. The samples are selected to represent all the production regions, and white and yellow maize, proportionally [12]. 

Approximately 350 wheat samples (depending on the season’s production) are annually selected as representative for the season’s wheat crop quality survey; from these, 40 wheat samples are selected for mycotoxin analyses [19].

The validated multi-mycotoxin LC-MS/MS method described in this study was used for the maize samples of four seasons from 2013–2014 to 2016–2017, and wheat samples of the four seasons from 2014–2015 to 2017–2018.

### 5.2. LC-MS/MS Mycotoxin Analysis

The LC-MS/MS multi-mycotoxin analytical method is an inhouse method developed and validated at the SAGL to be a fit-for-purpose, cost-effective method with acceptable limits of quantitation and a throughput of number of samples per day at an affordable cost/sample. This method is accredited for the analysis of multi-mycotoxins in grain, cereals and related feed samples according to ISO 17025 by the South African National Accreditation System (SANAS) [41,42].

#### 5.2.1. Sample Processing

The maize samples were milled with a Retsch ZM 200 mill with a 1-mm sieve, and the wheat samples with a hammer mill with a 0.8-mm sieve. The particle size of the milled sample was important to ensure complete extraction of the mycotoxins. It was also critical to ensure that the milled samples are thoroughly mixed, because small (10 g) subsamples were analyzed and the mycotoxins are heterogeneously distributed in the grain. Test results confirmed that a mixing time of at least 90 min with a mechanical mixer was needed to ensure a relative standard deviation (RSD) less than 20% in the aflatoxin B_1_ results of replicate analyses of 10-g subsamples at low µg/kg concentrations. 

#### 5.2.2. Chemicals, Standards and Reference Materials 

Methanol (MeOH, for HPLC, > 99.9%), acetonitrile (AcCN, ACS/HPLC grade, Burdick and Jackson), ammonium acetate (Purity ≥ 98%, Sigma-Aldrich/Merck), formic acid, (98%–100% Suprapur, Merck). Ultra-pure water (<18.2 MΩ.cm) was produced by a Milli-Q system (Millipore, Bedford, MA, USA).

Neat, solid mycotoxin standards (10 mycotoxins) and an HT-2 toxin calibrant solution (100 µg/mL) were purchased from Romer Labs Diagnostic GmbH (Tulln, Austria). Fumonisin B_2_ and fumonisin B_3_ were purchased from Cape Peninsula University of Technology (Cape Town, South Africa).

#### 5.2.3. Sample Preparation

Ten-gram subsamples (10.00 ± 0.05 g) were weighed and 40 mL extraction solution, 50:25:25 H_2_O:MeOH:AcCN (v/v) added. The samples were blended with an overhead stirrer for 1 min. The blended sample was transferred to a 50-mL polypropylene centrifuge tube and extracted for 15 min on a mechanical shaker in a horizontal position at 260 rpm. The extracted sample was centrifuged at 3000 rpm for 10 min. A 5-mL aliquot was pipetted into a volumetric flask and diluted by adding 5 mL of the dilution solution (25% MeOH in H_2_O (v/v)). The final sample extracts were filtered through 13-mm, 0.22-µm syringe filters (Nylon, Membrane Solutions) into the HPLC amber vials.

#### 5.2.4. LC-MS/MS Analysis

An ultra-performance liquid chromatograph (Waters Acquity UPLC) equipped with a C_18_ column (Acquity BEH, 1.7 µm, 50 × 2.1 mm i.d.) at 30 °C was used for the reverse phase chromatographic separation of the 13 mycotoxins with a programmed gradient elution of water containing 0.5 mM ammonium acetate and 0.1% formic acid (mobile phase A) and acetonitrile with 0.1% formic acid (mobile phase B) at a column flow rate of 0.4 mL and a run time of 15 min. 

The mass spectrometer was a Waters tandem quadrupole (Acquity TQD) equipped with an ESI source operating in positive ionization mode (ESI+) with a MassLynx v4.1 operating system. The optimized TQD parameters were source temperature (120 °C), desolvation temperature (500 °C), gas flow (N_2_, 650 L/hour) and cone gas flow (50 L/hour). Argon was used as collision gas in the collision cell. The cone voltages and collision energy values were optimized for each precursor ion. The M+1 ions were used for all the mycotoxins except the T2 and HT-2 toxins. The M + NH_4_ adduct ions were used as precursor ions for T2 and HT-2 toxins. One precursor ion and two product ions (MRMs) were optimized, selecting one product ion for quantification, and a second one for confirmation of the compound. 

Matrix-matched working standards, containing the 13 mycotoxins (Table 4), were prepared regularly for the calibration of the LC-MS/MS. 

### 5.3. Method Performance

#### 5.3.1. Method Validation

With the validation of the inhouse method, calibration curves of the matrix-matched standards (with at least five concentration levels) were compiled, using weighted linear regression (1/x). Linearity of the concentration ranges of each mycotoxin was confirmed with the evaluation of coefficients R^2^ and the calculation of the residuals. The validation results are summarized in Table 4. 

For the recovery and precision experiments, maize and wheat samples not contaminated with the analyzed mycotoxins were spiked with the mycotoxin working-standard mixture on at least three concentrations levels for each mycotoxin, including the limit of quantitation levels. Replicate recovery determinations (at least five) were conducted on each concentration level on different days using the described analytical method. The concentrations of the spiked samples were determined with the matrix-matched standards’ calibration curves. The percentage recovery (an indication of the trueness or accuracy of the method) was calculated as percentage recovery = µg/kg mycotoxin measured/µg/kg mycotoxin added × 100.

The precision (repeatability) was determined by calculating the RSD of the mycotoxin concentrations of the spiked samples at each concentration level and the average RSD of all the results/mycotoxin.

The limit of quantitation (LOQ) of each mycotoxin was established as the lowest concentration of the mycotoxin that was validated with acceptable accuracy and precision by applying the complete analytical method [43]. 

The percentage recoveries and precisions reported are well within the performance criteria established for the different mycotoxins according to EC Regulation No 401/2006, to which a method of analysis for the official control of mycotoxin levels in foodstuffs shall comply [6].

Certified reference materials for all regulated mycotoxins are not available, therefore the accuracy of the validated multi-mycotoxin method is confirmed by the continuous, successful participation in relevant proficiency testing schemes organized by FAPAS (Fera Science Ltd, UK) and BIPEA (France) analyzing grain and feed matrices. 

The uncertainty of measurement estimation for each mycotoxin, based on the EURACHEM/CITAC guide [44], was done with the whole method approach using the method validation data. All the sources of uncertainty were identified, and the three major contributions were precision, bias (recovery) and percentage purity of the reference standards. Their contributions were expressed as standard uncertainties, calculated from standard deviations. The standard uncertainties ranged from 10% to 20% and were in agreement with the maximum standard uncertainties specified in the “fitness-for-purpose” approach defined in EC Regulation No. 401/2006 [6]. The expanded uncertainties with a coverage factor of 1.96 ranged from 18% to 39% for each mycotoxin.

#### 5.3.2. Ongoing Method Performance Verification

The ongoing method performance verification procedures and acceptance criteria as described in SANCO/12571/2013 were followed [43]. All samples were analyzed in duplicate and the mean values were reported; the RSD of the duplicate results should not exceed 30% for concentrations significantly above the LOQ. Two spiked samples, one at a low and one at a higher concentration level, were included in every batch of samples and the practical acceptable recovery range of 60%–140% was applied. The results were not adjusted for recovery. 

## Figures and Tables

**Table 1:** Deoxynivalenol (DON) occurrence, mean and maximum concentration in SA commercial wheat samples collected after harvest in four consecutive production seasons.

Production Season	Number of Samples with DON	DON, Mean Concentration ^1^, µg/kg	DON, Maximum Concentration ^2^, µg/kg
2014–2015	5/40	229	361
2015–2016	4/40	397	593
2016–2017	4/40	289	501
2017–2018	7/40	202	570

^1^ Mean values based on positive samples. ^2^ Maximum values found in individual samples.

**Table 2:** Deoxynivalenol concentration range distribution in SA commercial post-harvest white and yellow maize.

DON Concentration Range, µg/kg	Percentage Samples with DON							
	White Maize				Yellow Maize			
	2013–2014	2014–2015	2015–2016	2016–2017	2013–2014	2014–2015	2015–2016	2016–2017
No deoxynivalenol (<LOQ = 100)	26.1	55.4	74.4	46.4	34.6	63.2	82.0	80.7
100 < 500	55.8	31.0	21.2	28.5	55.1	34.1	17.0	13.5
500–1000	10.3	7.7	3.8	7.3	8.1	2.7	1.0	3.5
> 1000–2000	6.7	4.2	0.6	8.9	1.6	0.0	0.0	2.3
> 2000 ^1^	1.2	1.8	0.0	8.9	0.5	0.0	0.0	0.0

^1^ 2000 µg/kg is the South African regulated maximum allowable DON level in unprocessed maize intended for human consumption [36].

**Table 3:** Fumonisin concentration range distribution in SA commercial post-harvest white and yellow maize.

FUM Concentration Range, µg/kg	Percentage Samples with FUM							
	White Maize				Yellow Maize			
	2013–2014	2014–2015	2015–2016	2016–2017	2013–2014	2014–2015	2015–2016	2016–2017
No fumonisins (<LOQ = 20)	56.4	42.3	38.5	55.3	61.6	45.1	46.9	56.1
20 < 750	37.6	47.6	51.9	40.8	31.9	46.2	40.2	36.3
750–2000	5.5	10.1	6.4	2.8	5.4	7.1	10.3	5.3
>2000–4000	0.6	0.0	2.6	1.1	0.5	1.6	1.5	1.2
> 4000 ^1^	0.0	0.0	0.6	0.0	0.5	0.0	1.0	1.2

^1^ 4000 µg/kg is the South African regulated maximum allowable FB_1_ + FB_2_ level in unprocessed maize intended for human consumption [36].

**Table 4:** Method validation results.

Mycotoxin	Recovery Range, %	Precision, Average RSD, %	Concentration Range of Spiked Samples, µg/kg	Limit of Quantitation (LOQ), µg/kg
Aflatoxin B_1_	76–106	11.4	2–100	2
Aflatoxin B_2_	77–108	12.8	2–100	2
Aflatoxin G_1_	79–114	12.2	2–100	2
Aflatoxin G_2_	88–118	15.4	5–100	5
Deoxynivalenol	80–93	13.2	100–400	100
15-Acetyl-deoxynivalenol	76–87	17.5	100–400	100
Fumonisin B_1_	72–94	19.6	20–400	20
Fumonisin B_2_	63–90	12.8	20–223	20
Fumonisin B_3_	68–87	13.4	20–204	20
Ochratoxin A	76–94	13.1	2–100	2
T2-Toxin	91–103	9.3	20–200	20
HT-2 toxin	79–105	12.1	20–200	20
Zearalenone	72–83	13.4	20–200	20

## Supplementary Materials

The following are available online at https://www.mdpi.com/2072-6651/11/5/271/s1, Table S1: Main mycotoxin results of SA commercial white maize (post-harvest) of four seasons from seven provinces, Table S2: Main mycotoxin results of SA commercial yellow maize (post-harvest) of four seasons from seven provinces.
