# Supplementary material for: Long Term Monitoring (2014–2018) of Multi-Mycotoxins in South African Commercial Maize and Wheat with a Locally Developed and Validated LC-MS/MS Method
Source: Toxins (Basel). 2019 May 14;11(5):271. doi: 10.3390/toxins11050271 (PMC6563230; doi:10.3390/toxins11050271)
Supplement: Supplementary file 1 [file toxins-11-00271-s001.pdf]

Supplementary Materials: Long term monitoring (2014-2018) of multi-mycotoxins in South African commercial maize and wheat with a locally developed and validated LC-MSMS method

Hannalien Meyer, Zanele Diana Skhosana, Mamsy Motlanthe, Wiana Louw and Egmont Rohwer

Table S1. Main mycotoxin results of SA commercial white maize (post-harvest) of four seasons from seven provinces.

Province	Season	Samples received	Tested samples		Positive samples[2]		Fumonisin $FB_1 + FB_2 + FB_3$			Deoxynivalenol			Zearalenone			FUM + DON[5]
		n[1]	n[1]	%	n[1]	%	n[1] positive	Mean[3], µg/kg	Max[4], µg/kg	n[1] positive	Mean[3], µg/kg	Max[4], µg/kg	n[1] positive	Mean[3], µg/kg	Max[4], µg/kg	n[1]
**Northern Cape**	2013-2014	0	-	-	-	-	-	-	-	-	-	-	-	-	-	-
	2014-2015	3	1	33	1	100	1	399	399	1	358	358	0	0	0	1
	2015-2016	2	1	50	1	100	1	790	790	1	110	110	0	0	0	0
	2016-2017	3	2	67	2	100	2	2015	3913	0	0	0	0	0	0	0
**North West**	2013-2014	89	33	37	31	94	23	296	758	24	435	1837	2	43	64	16
	2014-2015	192	53	28	47	89	46	333	1439	8	415	1391	1	93	93	8
	2015-2016	128	48	38	40	83	40	606	6865	5	315	598	0	0	0	2
	2016-2017	158	42	27	31	74	27	250	1441	18	849	7698	2	59	65	13
**Free State**	2013-2014	168	63	38	51	81	27	624	2927	38	259	791	2	54	82	14
	2014-2015	96	32	33	28	88	17	478	1727	12	1383	9736	5	121	337	3
	2015-2016	71	26	37	14	54	14	684	3430	7	505	1585	2	25	26	2
	2016-2017	168	54	32	30	56	13	230	836	25	445	1868	2	52	73	8
**Mpumalanga**	2013-2014	128	45	35	43	96	15	318	1195	41	596	2730	13	89	445	13
	2014-2015	128	60	47	48	80	18	316	1320	43	504	3260	12	63	212	14
	2015-2016	125	48	38	25	52	17	219	785	20	293	801	7	71	127	8

**Province**	**Season**	**Samples received**	**Tested samples**		**Positive samples**[2]		**Fumonisin $FB_1 + FB_2 + FB_3$**			**Deoxynivalenol**			**Zearalenone**			**FUM + DON**[5]
		**n**[1]	**n**[1]	**%**	n[1]	**%**	**n**[1] **positive**	**Mean**[3]**, µg/kg**	**Max**[4]**, µg/kg**	**n**[1] **positive**	**Mean**[3]**, µg/kg**	**Max**[4]**, µg/kg**	**n**[1] **positive**	**Mean**[3]**, µg/kg**	**Max**[4]**, µg/kg**	**n**[1]
	2016-2017	142	52	37	46	88	28	292	2135	37	1595	7671	13	121	399	17
**Gauteng**	2013-2014	39	13	33	11	85	3	161	418	11	964	6134	2	51	56	3
	2014-2015	38	12	32	12	100	9	353	1809	7	404	1135	2	33	35	4
	2015-2016	30	11	37	7	64	7	144	339	3	169	257	0	0		1
	2016-2017	39	14	36	10	71	3	264	708	9	1355	3157	4	59	117	2
**Limpopo**	2013-2014	7	3	43	3	100	2	376	394	1	444	444	0	0	0	0
	2014-2015	5	2	40	1	50	1	84	84	0	0	0	0	0	0	0
	2015-2016	27	10	37	8	80	8	293	854	0	0	0	0	0	0	0
	2016-2017	9	4	44	1	25	1	24	24	0	0	0	0	0	0	0
**KwaZulu Natal**	2013-2014	20	8	40	7	88	2	50	78	7	488	1351	2	44	47	2
	2014-2015	23	8	35	7	88	5	634	1575	4	722	1285	4	27	36	2
	2015-2016	32	12	38	10	83	9	192	426	3	197	253	1	65	65	2
	2016-2017	30	11	37	10	91	6	413	783	7	929	3836	3	70	128	3

[1] Number of samples. [2] Positive samples defined as concentration ≥ Limit of quantitation. [3] Mean concentration of positive samples. [4] Max = maximum concentration found in one individual sample. [5] FUM + DON = Samples with both FUM and DON.

Table S2. Main mycotoxin results of SA commercial yellow maize (post-harvest) of four seasons from seven provinces.

**Province**	**Season**	**Samples received**	**Tested samples**		**Positive samples**[2]		**Fumonisin FB1 + FB2 + FB3**			**Deoxynivalenol**			**Zearalenone**			**FUM + DON**[5]
		**n**[1]	**n**[1]	**%**	n[1]	**%**	**n**[1] **positive**	**Mean**[3], **µg/kg**	**Max**[4], **µg/kg**	**n**[1] **positive**	**Mean**[3], **µg/kg**	**Max**[4], **µg/kg**	**n**[1] **positive**	**Mean**[3], **µg/kg**	**Max**[4], **µg/kg**	**n**[1]
**Northern Cape**	2013-2014	16	5	31	5	100	4	752	1801	2	273	391	0	0	0	1
	2014-2015	24	9	38	7	78	6	206	779	2	435	567	0	0	0	1
	2015-2016	50	17	34	16	94	16	1257	7801	13	114	554	1	44	44	4
	2016-2017	41	14	34	14	100	14	825	2435	6	575	795	0	0	0	6
**North West**	2013-2014	160	59	37	52	88	16	383	1003	17	375	1353	3	35	44	11
	2014-2015	108	51	47	39	76	37	429	2200	9	210	328	2	43	60	7
	2015-2016	63	25	40	20	80	19	1158	11347	2	137	159	0	0	0	1
	2016-2017	39	27	69	20	74	16	305	1244	10	537	1488	1	113	113	6
**Free State**	2013-2014	305	114	37	80	70	18	478	1720	20	255	868	0	0	0	10
	2014-2015	77	29	38	26	90	19	658	3382	12	242	528	1	23	23	5
	2015-2016	81	32	40	14	44	14	334	1109	2	507	640	0	0	0	1
	2016-2017	116	45	39	14	31	12	265	847	2	146	148	0	0	0	1
**Mpumalanga**	2013-2014	331	125	38	113	90	24	518	5357	65	396	2601	13	103	354	20
	2014-2015	249	71	29	50	70	27	380	1236	34	346	851	7	42	71	13
	2015-2016	214	80	37	37	46	29	446	1993	16	164	304	3	35	36	5
	2016-2017	177	59	33	29	49	24	773	6059	12	398	1552	1	24	24	4
**Gauteng**	2013-2014	69	25	36	22	88	5	482	1429	10	292	825	1	27	27	4
	2014-2015	27	11	41	4	36	3	433	652	3	199	287	0	0	0	2
	2015-2016	31	13	42	7	54	6	607	1431	1	100	100	0	0	0	1
	2016-2017	40	14	35	4	29	4	646	1307	2	227	321	0	0	0	2
**Limpopo**	2013-2014	15	6	40	6	100	2	1332	2482	2	252	350	0	0	0	1

**Province**	**Season**	**Samples received**	**Tested samples**		**Positive samples**[2]		**Fumonisin $FB_1 + FB_2 + FB_3$**			**Deoxynivalenol**			**Zearalenone**			**FUM + DON**[5]
		**n**[1]	**n**[1]	%	n[1]	%	**n**[1] **positive**	**Mean**[3], **µg/kg**	**Max**[4], **µg/kg**	**n**[1] **positive**	**Mean**[3], **µg/kg**	**Max**[4], **µg/kg**	**n**[1] **positive**	**Mean**[3], **µg/kg**	**Max**[4], **µg/kg**	**n**[1]
	2014-2015	6	2	33	2	100	2	146	210	0	0	0	0	0	0	0
	2015-2016	16	6	38	5	83	5	492	1086	0	0	0	0	0	0	0
	2016-2017	10	3	30	2	67	2	57	75	0	0	0	0	0	0	0
**KwaZulu Natal**	2013-2014	34	16	47	12	75	2	95	163	5	180	236	2	80	127	2
	2014-2015	24	9	38	8	89	6	149	331	7	183	372	3	65	124	5
	2015-2016	39	16	41	11	69	10	375	863	4	163	234	1	23	23	3
	2016-2017	28	9	32	4	44	3	235	453	1	130	130	0	0	0	0

[1] Number of samples. [2] Positive samples defined as concentration ≥ limit of quantitation. [3] Mean concentration of positive samples. [4] Max = maximum concentration found in one individual sample. [5] FUM + DON = Samples with both FUM and DON.
